# Supplementary material for: Evaluation and Monitoring of the Natural Toxin Ptaquiloside in Bracken Fern, Meat, and Dairy Products
Source: Toxins (Basel). 2023 Mar 21;15(3):231. doi: 10.3390/toxins15030231 (PMC10053987; doi:10.3390/toxins15030231)
Supplement: Supplementary file 1 [file toxins-15-00231-s001.zip › toxins-2223897-supplementary.pdf]

Supplementary Materials

Table S1. Multiple reaction monitoring (MRM) parameters and retention time for target compound.

Analyte	Monoisotopic mass (g/mol)	Precursor ion (*m*/*z*)	Ionization state	Product ion (*m*/*z*)	Collision energy (eV)	Retention time (min)
				181*	16	
Ptaquiloside	398.1	399	$[M + H]^+$	381	10	3.21
				277	10	

*Underlined ion is quantitative ion, and other ions are qualitative ions.

Table S2. Detailed LC–MS parameters.

Parameter	Conditions
Liquid chromatograph<br>Mass spectrometer	Vanquish UHPLC (Thermo Fisher Scientific, California, USA)<br>TSQ Altis (Thermo Fisher Scientific, California, USA)
Column	Kinetex 2.6-µm C18 100 Å (100 × 21 mm²)
Mobile Phase	A: Water with 0.1% of formic acid (v/v)<br>B: Acetonitrile with 0.1% of formic acid (v/v)
Flow rate	0.5 mL/min
Injection volume	10 µL
Column temperature	35 °C
Ion mode	ESI positive
The gradient elution condition	5% B (0–0.5 min), 5%–95% B (0.5–3 min), 95% B (3–4 min), 95%–5% B (4–4.1 min), and 5% B (4.1–5 min)
Ion Source condition	Ion spray voltage: 3500 V, sheath gas: 50 Arb, aux gas: 10 Arb, sweep gas: 1 Arb, ion transfer–tube temperature: 325 °C, vaporization temperature: 350 °C, and dwell time: 48 ms
MRM transition	Quantitative ion pair: *m*/*z* 399 > 181 (CE: 16 eV)<br>Confirmatory ions pairs: *m*/*z* 399 > 381 (CE: 10 eV) and *m*/*z* 399 > 277 (CE: 10 eV)

Table S3. Ptaquiloside (PTA) calibration curve equation, linearity, the limit of detection (LOD), and limit of quantification (LOQ).

Range (µg/kg)	Equation (y = ax + b)	Linearity (r^2)	LOD (µg/kg)	LOQ (µg/kg)
0.1–50	y = 37732x + 1079.4	0.9979	0.03	0.09

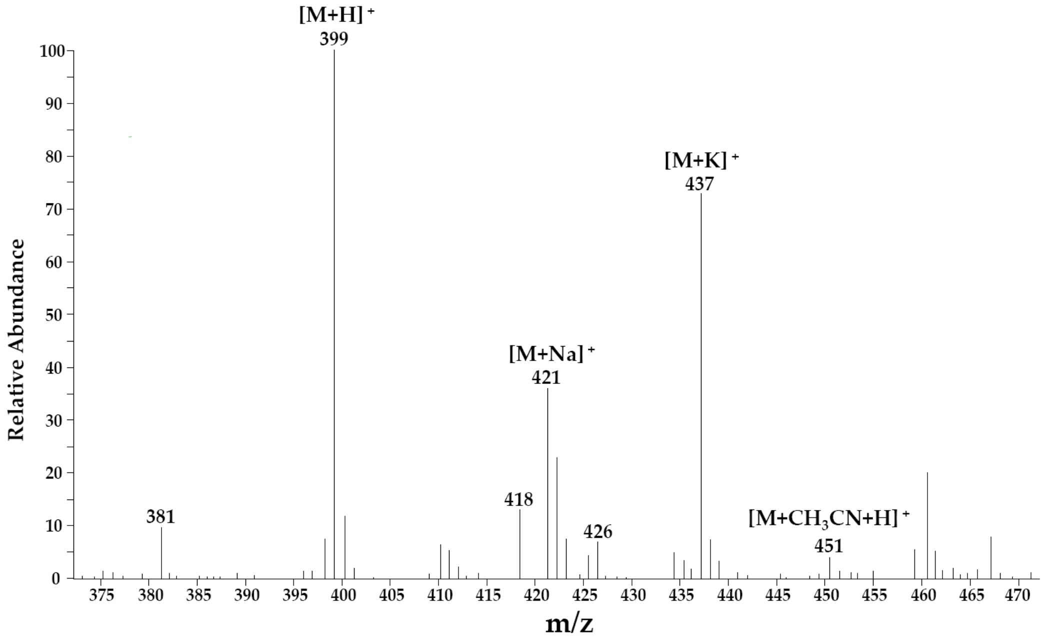

Figure S1. Q3 full scan of ptaquiloside (PTA) standard working solution.

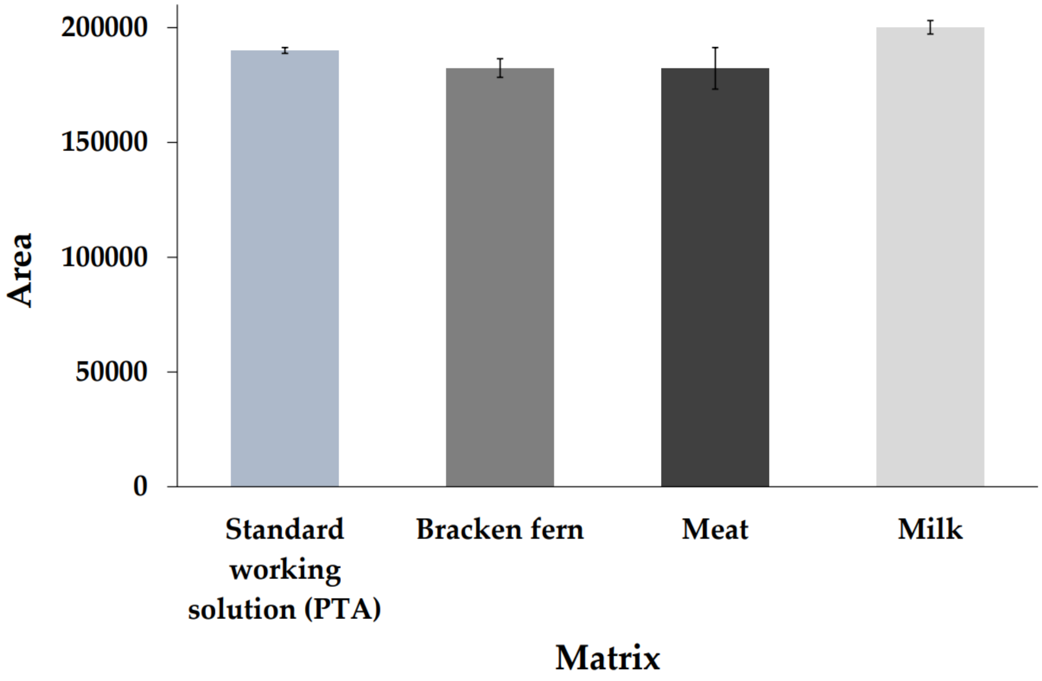

Matrix	The area value difference (%) with the PTA standard working solution [a]
Bracken fern	− 4.0
Meat	− 4.1
Milk	+ 5.3

a: The area value difference (%) with the PTA standard working solution = [(the area value of PTA standard working solution − the area value PTA spiked into each matrix) / the area value of PTA standard working solution] × 100 ($n = 3$, and error bars represent standard deviation)

Figure S2. Comparison of the relative differences in the peak areas for the same concentration of (20 μg/kg) analyte (ptaquiloside; PTA) in different matrices (bracken fern, meat, and milk) and the standard working solution for PTA.

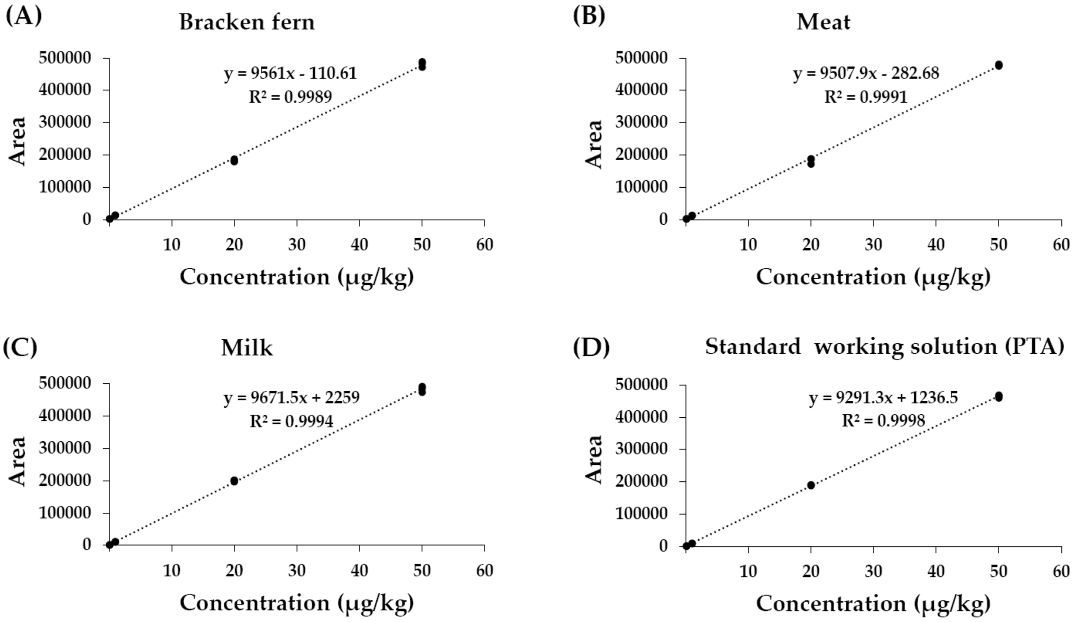

Figure S3. A procedure for verifying the relative matrix effect in different matrices: bracken fern (A), meat (B) milk (C), and PTA standard working solution (D).

Table S4. Comparison of the slope of each matrix-matched standard calibration curve with the slope of the PTA standard calibration curve.

Matrix	Equation (y = ax + b)	Linearity (r^2)	Slope difference (%)[a]	Slope CV (%)[b]
Bracken fern	y = 9561x − 110.61	0.9989	2.9	
Meat	y = 9507.9x − 282.68	0.9991	2.3	
Milk	y = 9671.5x + 2259	0.9994	4.1	1.7
Standard working solution (PTA)	y = 9291.3x + 1236.5	0.9998	-	

a: [(Slope value of standard working solution (PTA) calibration curve − the slope values of each matrix-matched standard calibration) / slope value of standard working solution (PTA)] × 100.
b: Precision value (coefficient of variation; CV, %) = [Standard deviation for the slope values of each matrix-matched calibration curve/Mean for the slope values of each matrix-matched calibration curve] × 100.

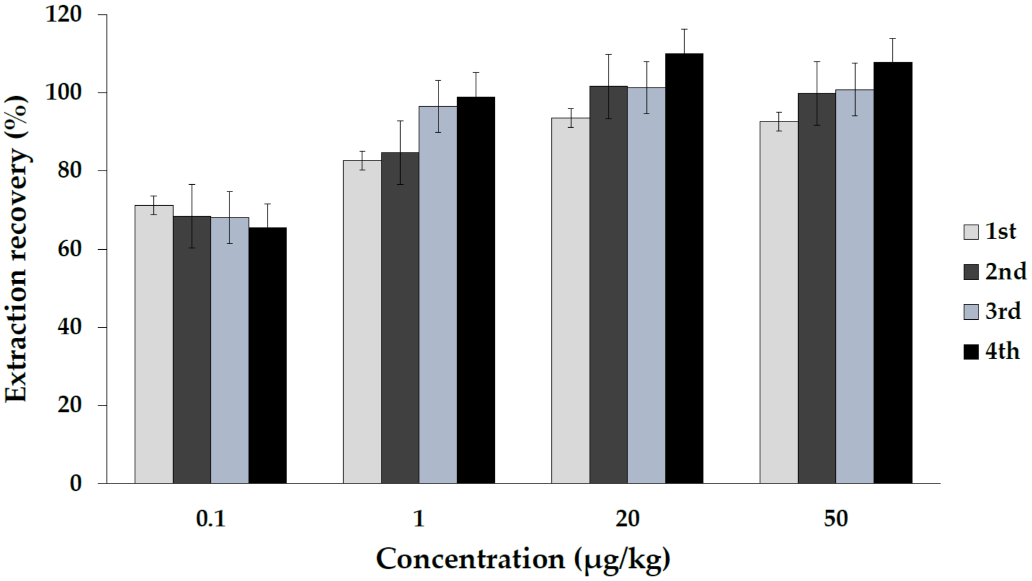

Figure S4. Extraction recovery (%) at 0.1, 1, 20, and 50 (μg/kg) levels of PTA. Measurements were 4 replicates for each concentration ($n = 3$, and *the error bars indicate the standard deviation of three samples per assay batch for each concentration*). In total, there were 4 ×4 × 3 = 96 samples analyzed. Extraction recovery (%) = (matrix spiked before extraction / matrix spiked after extraction) × 100.
